# Automaticity of walking: functional significance, mechanisms, measurement and rehabilitation strategies

**DOI:** 10.3389/fnhum.2015.00246

**Published:** 2015-05-05

**Authors:** David J. Clark

**Affiliations:** ^1^Brain Rehabilitation Research Center, Malcom Randall VA Medical Center, North Florida/South Georgia Veterans Health SystemGainesville, FL, USA; ^2^Department of Aging and Geriatric Research, University of FloridaGainesville, FL, USA

**Keywords:** walking, motor control, near infrared spectroscopy, rehabilitation, automaticity, executive control, dual task

## Abstract

Automaticity is a hallmark feature of walking in adults who are healthy and well-functioning. In the context of walking, “automaticity” refers to the ability of the nervous system to successfully control typical steady state walking with minimal use of attention-demanding executive control resources. Converging lines of evidence indicate that walking deficits and disorders are characterized in part by a shift in the locomotor control strategy from healthy automaticity to compensatory executive control. This is potentially detrimental to walking performance, as an executive control strategy is not optimized for locomotor control. Furthermore, it places excessive demands on a limited pool of executive reserves. The result is compromised ability to perform basic and complex walking tasks and heightened risk for adverse mobility outcomes including falls. Strategies for rehabilitation of automaticity are not well defined, which is due to both a lack of systematic research into the causes of impaired automaticity and to a lack of robust neurophysiological assessments by which to gauge automaticity. These gaps in knowledge are concerning given the serious functional implications of compromised automaticity. Therefore, the objective of this article is to advance the science of automaticity of walking by consolidating evidence and identifying gaps in knowledge regarding: (a) functional significance of automaticity; (b) neurophysiology of automaticity; (c) measurement of automaticity; (d) mechanistic factors that compromise automaticity; and (e) strategies for rehabilitation of automaticity.

## Introduction

Safe and independent mobility function at home and in the community requires well-coordinated control of walking. A hallmark of this healthy control of walking is automaticity, which is the ability of the nervous system to successfully coordinate movement with minimal use of attention-demanding executive control resources. The term “automaticity” is fairly common in literature about control of walking (for example Paul et al., 2005; Hallett, 2008; Bridenbaugh and Kressig, 2011; Fasano et al., 2012). However, it often defined loosely and presented in a theoretical context rather than as a tangible property of locomotor control that can be evaluated and intervened upon. This is a potential oversight that may be detrimental to achieving optimal recovery of mobility function in a variety of clinical populations. Accordingly, this review article seeks to consolidate evidence from multiple domains of neuroscience and rehabilitation in order to advance the science of automaticity of walking. This will fill a gap in the literature by providing a unifying discussion of automaticity that spans the topics of functional significance, neurophysiological determinants, measurement, mechanisms of impairment, and strategies for rehabilitation.

## Functional Significance of Automaticity vs. Executive Locomotor Control

Control of walking is seldom, if ever, purely under the control of either automatic or executive control processes. Rather, there is a balance between the two processes that is dependent upon the demands of the task and the capabilities of the individual. This balance has extremely important implications for the efficacy and safety of task performance. Research by Shiffrin, Schneider and colleagues provides a framework for understanding the important functional implications of automaticity (Schneider and Shiffrin, 1977; Shiffrin and Schneider, 1977). This framework was developed to explain two complementary forms of cognitive processing, automatic and controlled, but the concepts can also be applied to locomotor control. These researchers defined automatic cognitive processing as the activation of a sequence of nodes that nearly always becomes active in response to a particular input configuration and that is activated automatically without the necessity for attention by the individual (Schneider and Shiffrin, 1977; Shiffrin and Schneider, 1977). Although the neural structures/networks underlying automatic cognitive processing differ from those underlying automatic locomotor control (see Section Neurophysiology of Automaticity), the two can be viewed as conceptually analogous. The opposite of automatic processing is controlled cognitive processing, which was defined as a temporary sequence of nodes activated under control of, and through attention by, the individual (Schneider and Shiffrin, 1977). Controlled processes are capacity limited, but the costs of this capacity limitation are balanced by the benefit of being set up, altered, and applied in novel situations for which automatic sequences have never been learned (Schneider and Shiffrin, 1977). In the present paper on locomotor control, the broader term “executive control” is used in place of “controlled processing”. There are a number of important phenomena that characterize the difference between automatic and controlled processing and that should be considered in the context of walking performance and safety. The first phenomenon is that automatic processing is fast and parallel, while controlled/executive processing is slow and serial (Schneider and Chein, 2003). In the context of walking, the use of an executive control strategy is concerning because it is less suited for managing the complexities of multi-joint movements in real time. For instance, crucial information from the periphery, such as unexpected changes in the slope or texture of the walking surface, must be quickly and accurately integrated into the ongoing gait cycle for safe ambulation. With automaticity of control, this information can be quickly delivered and integrated via spinal reflex pathways (i.e., fast, parallel processing of information) (Zehr and Stein, 1999; af Klint et al., 2008). In contrast, an executive control strategy would require a much longer time period for peripheral information to be delivered and processed in the cerebrum before subsequent integration with the gait pattern (i.e., slow, serial processing of information). Furthermore, the resultant neural commands may be less appropriate and more variable. A second phenomenon is that automatic processing requires little effort and can operate in high workload situations, whereas controlled/executive processing requires substantial effort and interferes with other controlled processing tasks (Schneider and Chein, 2003). This is concerning for walking because loss of automaticity and a compensatory reliance on executive control could overly encumber the available supply of executive resources. This will lead to a competition for executive resources and may result in performance decrements for walking and concurrent tasks (Ojha et al., 2009; Clark et al., 2014b). Such a decrement is commonly referred to as the “cost” of multi-tasking. This issue has also been described as a “supply and demand problem”, such that the cumulative demand for executive control resources exceeds the available supply (Seidler et al., 2010). A sufficient supply of executive resources is important for walking performance under complex environmental conditions (Clark et al., 2014b). For example consider the demands of walking in a crowded shopping mall. If the executive resources needed for this task are encumbered by the control of the basic walking pattern, there is a heightened risk that hazards may be overlooked or ignored. The individual may be less likely to notice a slick puddle on the floor or may misjudge the speed or direction of surrounding pedestrians, resulting in slips, trips, collisions and falls. A third phenomenon is that automatic processing is far less sensitive to stressors than is performance under controlled/executive processing (Schneider and Chein, 2003). This implies that environmental conditions that are challenging or anxiety-provoking may substantially deteriorate performance of executive locomotor control. One example is the challenge/anxiety associated with walking across a busy street. Dommes and colleagues suggest that the attentional demands of gait and balance in older adults may contribute to instances of poor decision making and dangerous behaviors during simulated street crossing. Specifically, older participants were found to cross more slowly, adopt smaller safety margins, and make more decisions that led to collisions than did young participants (Dommes et al., 2014). The cumulative evidence indicates that compromised automaticity of walking has important functional implications, which highlights the crucial need for improved mechanistic understanding and enhanced rehabilitative strategies.

## Neurophysiology of Automaticity

Automaticity of walking is made possible by specialized circuits in the central nervous system (CNS) that are capable of coordinating complex patterns of neuromuscular activation. The circuits have been fine-tuned over millions of years of evolution (Nielsen, 2003) to allow for a stable yet flexible locomotor control strategy that does not require continuous attentional control. The most well-described circuits (primarily revealed by animal studies of locomotor control) are located in the spinal cord, brainstem and cerebellum. This section will briefly describe some of these major neural circuits.

The “central pattern generator” circuits of the spinal cord are perhaps the most well-known locomotor circuits supporting automaticity. Evidence from animals and humans reveals that non-patterned electrical input to the lumbar spinal cord can elicit flexion/extension movements of the limbs that are similar to walking, even in the absence of input from the brain (Grillner, 1981). For instance, Dimitrijevic and colleagues used epidural stimulation of the posterior spinal cord to elicit locomotor-like limb movements in adults with complete spinal cord injury. This finding complements earlier research that demonstrated the ability of decerebrate cats to perform basic stepping movements (Sherrington, 1910; Brown, 1911). Spinal pattern generating circuits may already be operational at birth, as they have been proposed to be responsible for coordinated kicking movements in human infants, as well as the “step reflex” that occurs when infants are stood upright with body weight supported (Forssberg, 1985). With maturation and practice, these circuits become more complex in order to facilitate coordinated adult locomotion (Ivanenko et al., 2004; Clark et al., 2010; Dominici et al., 2011). At the next level of the neuraxis are brainstem circuits of locomotor control. Electrical stimulation of isolated brainstem regions has been shown to evoke walking-like behaviors. The two key regions that have been identified are the mesencephalic locomotor region (MLR) and subthalamic locomotor region (SLR). The MLR has been observed in all vertebrate species tested to date, including lamprey, salamander, stingray, rat, guinea-pig, rabbit, cat, and monkey (Le Ray et al., 2011; Ryczko and Dubuc, 2013). It provides excitatory input to the spinal cord that serves to initiate, scale, and sustain the descending command for walking (Le Ray et al., 2011; Ryczko and Dubuc, 2013). The SLR is considered to be closely related to the MLR and has been found in a number of vertebrates including rats and cats (Kasicki et al., 1991; Narita et al., 2002). It may have particular relevance for scaling locomotor output, such as when inducing changes in speed and cadence (e.g., walking vs. running) (Narita et al., 2002). In addition to brainstem locomotor regions, a cerebellar locomotor region has been reported in cats (Mori et al., 1998). Furthermore, studies in humans with cerebellar damage have shown the important role of the cerebellum in the control and coordination of balance and walking (Morton and Bastian, 2004). Among the notable findings with cerebellar damage are ataxic gait, impaired motor learning, and compromised ability to make predictive gait and balance modifications (Horak and Diener, 1994; Morton and Bastian, 2004, 2006). Finally, descending excitatory drive from cerebral motor pathways is considered crucial to facilitating the brainstem and spinal circuits of automaticity in humans (Yang and Gorassini, 2006). Emerging evidence from studies using electroencephalography and transcranial magnetic stimulation further suggest a direct involvement of motor cortex in driving muscle activation, even during undemanding steady state walking (Petersen et al., 2001, 2012). Accordingly, some aspects of automaticity of walking may reside in cerebral circuits. Cumulatively, the CNS circuits discussed here comprise the neurophysiological architecture that allows for automaticity of walking without the need for continuous attentional monitoring and executive control.

## Measuring the Balance Between Automatic and Executive Control of Walking

A challenge to studying automaticity is that the CNS circuits cannot be directly assessed in humans. Rather, the balance between automaticity and executive control must be inferred from assessments that gauge heightened utilization of an executive control strategy during walking. The underlying premise is that, during undemanding steady state walking, executive control is used as a compensatory control strategy in the absence of robust automaticity. Executive control involves the use of attentional and intentional resources in the cerebrum to monitor and execute movements. The most widely used approach for probing automatic vs. executive control is assessment of dual-tasking. This is a behavioral approach in which a single task of interest, such as walking, is performed alone (single-task) as well as simultaneously with another task (dual-task). Often, the dual-task condition yields a decrement in performance compared to the single task condition. The size of the decrement, called the “dual-task cost”, is interpreted to result from a competition for executive control resources. When the single task requires heightened executive control, the dual-task cost is expected to be higher. In contrast, automatic control of the single task is expected to yield a lower dual-task cost. Although the premise is fairly simple, in reality the determinants of dual-task cost are multi-factorial and potentially complicated. The instructions given to the participant, particularly with regarding to task prioritization, are known to substantially influence the results. Furthermore, the difficulty level of the secondary task (often verbal fluency or mathematical problem solving) varies greatly based on the task chosen and the capabilities of the individual being tested. Prior articles have provided substantial discussion and review of dual-tasking assessments (Beauchet and Berrut, 2006; Beauchet et al., 2009; Plummer et al., 2013; Patel and Bhatt, 2014).

Neurophysiological assessments offer an alternative approach to dual-tasking for measuring the balance between automaticity and executive control of walking. Among the most promising is functional near infrared spectroscopy (fNIRS), because it provides continuous, noninvasive, unobtrusive monitoring and can be used in ecologically valid settings including during walking (Ayaz et al., 2012b; Holtzer et al., 2014; Perrey, 2014; Piper et al., 2014). The major drawback of fNIRS is that it is limited to superficial recording of cortex and has lower spatial resolution than functional magnetic resonance imaging. During fNIRS assessment, a laser diode at the surface of the skin emits near-infrared light which passes through soft tissue and bone to reach the cerebral cortex. In the cortex, some near-infrared light is absorbed by hemoglobin while a proportion of the non-absorbed light scatters back to the surface. This non-absorbed light is then measured by a highly sensitive photodiode. Because oxyhemoglobin and deoxyhemoglobin preferentially absorb light of different wavelengths, the concentration changes of oxy- and deoxy-hemoglobin can be calculated. Hemoglobin concentrations are directly affected by metabolic activity in cortical tissue and the resultant changes in blood flow. In addition to fNIRS, other assessment approaches also show great promise, including positron emission tomography, electroencephalography, frequency-based analysis of electrophysiological signals, and fMRI during imagined walking (Duckrow et al., 1999; Cham et al., 2008; Shoushtarian et al., 2011; Petersen et al., 2012; Clark et al., 2013; Shimada et al., 2013; Holtzer et al., 2014). Most of the relevant literature that is cited in the present article uses fNIRS as the neurophysiological assessment.

For monitoring the use of executive control resources, an important brain region is prefrontal cortex. The prefrontal cortex operates at the highest levels of the control hierarchy, contributing to a cascade of processes that mediate task planning and execution for cognitive and motor functions (Koechlin et al., 2003; Parasuraman and Caggiano, 2005; Bear et al., 2007). It plays an essential role as an interface between cognition, action, and the physical world (Derosière et al., 2013). The literature reports that prefrontal cortical activity is heightened during the performance of cognitive tasks (Herrmann et al., 2006; Kaneko et al., 2011; Ohsugi et al., 2013), fine motor tasks (Okamoto et al., 2004), and dual-tasks (Holtzer et al., 2011; Doi et al., 2013; Ohsugi et al., 2013). A number of studies have detected incremental increases in parallel with the complexity of cognitive tasks (Shibuya-Tayoshi et al., 2007; Kaneko et al., 2011; Ayaz et al., 2012b; Verner et al., 2013). Likewise, a number of fNIRS studies of walking have demonstrated that more complex walking tasks also require heightened prefrontal activity relative to undemanding steady state walking. For example, prefrontal activity is significantly elevated when preparing for gait initiation or executing speed changes (Suzuki et al., 2004, 2008; Mihara et al., 2007; Clark et al., 2014b), during the performance of complex walking tasks that require careful control of posture and of limb movements (Atsumori et al., 2010; Clark et al., 2014b; Koenraadt et al., 2014), and during dual-task walking (Clark et al., 2014b; Meester et al., 2014). In contrast, walking at different steady state walking speeds (e.g., slow speed vs. moderate speed) does not substantially affect prefrontal activation (Suzuki et al., 2004; Meester et al., 2014). This latter finding is presumably due to the ability of brainstem and spinal circuits of automaticity to alter the rate of locomotor pattern generation without the need for substantial executive control resources. Heightened prefrontal activity may also reflect an increased executive demand to compensate for loss of automaticity due to neurological or peripheral (e.g., musculoskeletal) impairments (Seidler et al., 2010) or due to impairment of CNS circuits of automaticity. Indeed, prefrontal activity has been shown to be elevated during steady state walking in the elderly, especially older adults with poorer gait performance (Harada et al., 2009) and those with ataxic gait (Mihara et al., 2007; Caliandro et al., 2012).

Prefrontal fNIRS assessment has also shown potential for explaining differences in functional task performance, including over the course of motor learning during acquisition of automaticity for a novel task. Ayaz and colleagues provide an excellent example of a transition from executive to automatic control with task practice. They measured the behavioral and neurophysiological responses of novice participants while they practiced the complex cognitive/motor task of piloting a virtual unmanned aerial vehicle in a flight simulator (Ayaz et al., 2012a,b). With practice, all measures indicated improvement in performance when comparing across beginner, intermediate and advanced phases of training. Analysis of change across days revealed that behavioral measures of flight performance provided more detailed information than subjective self-reported measures. Furthermore, changes in prefrontal activation measured by fNIRS provided even more detail. Specifically, at the beginner level, behavioral performance improved each day at the cost of heightened prefrontal activation (i.e., heightened executive demand). This suggests that increased effort was required to learn the skill. In the intermediate phase, a higher level of behavioral performance could be maintained with less prefrontal activation. Finally, in the advanced phase, an even higher level of behavioral performance was achieved with a trend toward a further reduction of prefrontal activation (Ayaz et al., 2012a). It is reasonable to expect that similar acquisition of automatic control could be attained with rehabilitation of walking.

Also supporting the use of prefrontal fNIRS to gauge the link between function and automaticity is work by Harada and colleagues, who compared prefrontal activity and driving performance in less experienced vs. more experienced drivers (Harada et al., 2007). Young adults who are less experienced exhibited a larger increase in prefrontal activity during driving, suggesting less automaticity. These less experienced drivers also exhibited unsafe driving behaviors such as not observing the door mirror carefully when changing lanes. This observation may be indicative of competing demand for executive control resources. Little research has been done thus far to link prefrontal activity to walking performance (Clark et al., 2014b), so this will be an important area for future research.

The interpretation of prefrontal activity should take into account the potential confounding effects of underlying physiological factors. For instance, fNIRS activity has been shown to be affected by the volume of underlying gray matter (Maillet and Rajah, 2013) and the health of the cerebrovascular and cardiovascular systems (Suhr and Chelberg, 2013). These factors are often compromised in older adults, for example. Similarly, individuals who are disengaged or unmotivated during performance of experimental tasks may exhibit smaller changes in cortical activity due to lower utilization of executive resources. Furthermore, some research has reported that prefrontal fNIRS may lack the sensitivity for detecting subtle changes in executive control (Derosière et al., 2013). Although much of the existing research that has used fNIRS during walking has assessed prefrontal cortex, it should be acknowledged that this is not the only cortical region that is likely to be involved in executive locomotor control. Indeed, prior fNIRS studies have shown walking-related changes in motor and somatosensory cortical regions (Suzuki et al., 2004, 2008; Kurz et al., 2012; Koenraadt et al., 2014). It will be important for future research to assess a broader array of cortical regions.

## Mechanistic Factors That Influence Automaticity

**Figure 1:**
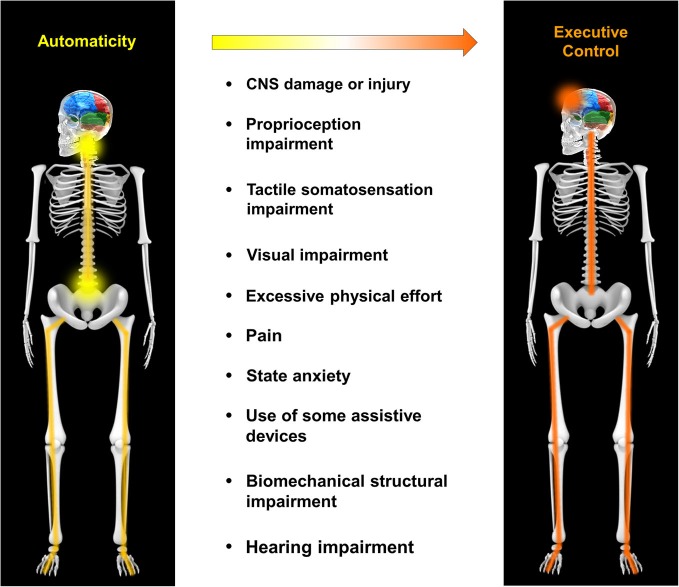
**Mechanistic factors that compromise automaticity of walking**. A variety of factors may contribute to a shift in the balance of locomotor control from automaticity to executive control. These include, but are not limited to central nervous system (CNS) damage/injury, proprioception impairment, tactile somatosensation impairment, visual impairment, excessive physical effort, pain, state anxiety, use of some assistive devices, biomechanical structural impairment and hearing impairment.

In the section entitled Neurophysiology of Automaticity, the neural circuits supporting automaticity of walking were discussed. This section will further expand upon mechanistic factors that may compromise automaticity by influencing the operation of those circuits, either directly or indirectly. The factors discussed include CNS injury/disease, proprioception, tactile somatosensation, visual impairment, physical effort, pain, state anxiety, use of assistive devices, biomechanical structure and hearing impairment (Figure 1). This should not be considered a definitive or all-inclusive list, but rather focuses on factors that are potentially important to clinical populations with compromised walking function. The order in which each factor is presented is roughly based on the strength of evidence supporting an effect on circuits of automaticity or on the balance between automatic and executive control of walking.

### Nervous System Damage/Injury

Damage or disease of the CNS can be devastating to walking function, as demonstrated by conditions such as stroke, spinal cord injury, Parkinson’s disease and others. The effect of CNS damage on automaticity may be due to a number of different factors. One is direct damage to CNS circuits of automaticity, such as may occur with injury to the lumbar spinal cord, or stroke affecting the brainstem. A second factor is disruption of facilitatory drive to circuits of automaticity, such as due to cortical stroke or injury to the upper spinal cord. A third factor is impairment of peripheral nervous system structure or function, such as sensory inputs like proprioception or vision. These and other impairments affecting automaticity are discussed below. The implications for automaticity by damage to any particular structure and its associated pathways will depend on the specific case. Walking assessments of people with CNS deficits are generally consistent with impairment of automaticity and heightened executive locomotor control, including poor dual task performance and heightened activity of prefrontal cortex during walking (Hyndman et al., 2006; Mihara et al., 2007; Dennis et al., 2009; Plotnik et al., 2009; Caliandro et al., 2012; Plummer-D’Amato and Altmann, 2012; Smulders et al., 2012; Panyakaew and Bhidayasiri, 2013).

### Proprioception

Proprioception provides input to the CNS about limb position and weight bearing, and is a crucial input for automaticity. Muscle spindles and Golgi tendon organs are proprioceptive sensory receptors that supply feedback about muscle length and musculotendinous force, respectively (Prochazka, 1981; Jami, 1992). This information plays an important role in triggering the initiation and maintenance of muscle activity that produce key events in the gait cycle (Dietz, 1996; Pearson, 2008). For instance, proprioceptive information induced by treadmill movement is sufficient for producing coordinated locomotor movements, even in decerebrate cats that lack descending control from the brain (Dimitrijevic et al., 1998; Grillner et al., 2008). Furthermore, removal of proprioceptive input by deafferentation reduces the magnitude of knee and ankle extensor muscle activity by approximately 70% (Hiebert and Pearson, 1999). Proprioceptive information from the hip joint and associated musculature is known to be important for appropriate control of gait biomechanics in humans and animals (Andersson and Grillner, 1981; Dietz et al., 2002). Hip extension during late stance phase of walking contributes to the initiation of swing phase (Hiebert et al., 1996; McVea et al., 2005), and mechanical perturbation of the limb during swing phase alters hip flexor activity (Lam and Pearson, 2001). Abnormal proprioceptive input to the CNS, such as due to impaired proprioception and/or abnormal walking patterns (e.g., poor hip kinematics which is common in clinical populations (Lee et al., 2005; Svehlik et al., 2009; Hyngstrom et al., 2010)), may significantly compromise automaticity of walking.

### Tactile Somatosensation

Sense of touch and vibration in the lower extremities is known to be a crucial factor that interacts with the central circuits of automaticity. Fallon and colleagues reported that information from skin mechanoreceptors on the sole of the foot exert a strong facilitation of spinal motorneuronal activity in the lower limbs (Fallon et al., 2005). Furthermore, cutaneous stimulation during walking in animals and humans has been shown to induce phase-specific modulation of limb movements (Frigon and Rossignol, 2006). Clinical research has consistently shown that decrements in tactile perception are strongly associated with compromised performance on tests of walking and balance (Resnick et al., 2000; Mold et al., 2004; Deshpande et al., 2008; Buchman et al., 2009; Cruz-Almeida et al., 2014). Although loss of automaticity cannot be directly implicated in this association, an interesting study by Paul and colleagues (Paul et al., 2009) provides some support for such an assertion. They show that dual-tasking ability is more impaired in older adult diabetics with peripheral neuropathy compared to older adult diabetics without peripheral neuropathy (Paul et al., 2009). This finding implies that peripheral impairments necessitate an increased demand and competition for executive control of walking, consistent with a lack of automaticity. If impaired tactile perception compromises automaticity, can augmenting tactile input enhance automaticity? This question was recently examined in research conducted by Clark and colleagues, who found that wearing textured insoles can reduce prefrontal cortical activation during walking in older adults with mild somatosensory deficits (Clark et al., 2014a). Less prefrontal activity implies a lower demand for executive control and thus a more automatic strategy of locomotor control. Furthermore, this finding offers a potential mechanistic explanation (i.e., enhanced automaticity) for numerous prior observations of improved static and dynamic balance when wearing textured or vibrating insoles (Priplata et al., 2003; Palluel et al., 2008, 2009; Qiu et al., 2012; Lipsitz et al., 2015).

### Visual Impairment

Visual information is a crucial sensory input that facilitates safe walking. A number of studies have shown associations between diminished or abnormal visual input and decrements in walking performance, including during control of steady state walking (Helbostad et al., 2009; Swenor et al., 2014) and during more complex tasks like obstacle crossing and curb negotiation (Alexander et al., 2014a,b; Novak and Deshpande, 2014). This may be due in part to impaired automaticity of walking. In one study it was shown that reduced visual input due to dim lighting yields an increase in prefrontal cortical activity during steady state walking, suggesting a shift in the balance from automatic to executive control (Clark et al., 2014b). Similarly, dual-task cost in Parkinson’s patients was found to be exacerbated by walking in dim lighting (Pieruccini-Faria et al., 2014). It has also been reported that the magnitude of mental effort required for mobility covaries with the severity of visual impairment in patients with retinitis pigmentosa (Turano et al., 1998). Accordingly, lack of visual information may be an important factor leading to compromised automaticity of walking.

### Physical Effort

Evidence suggests a shift in the balance from automaticity to executive control for tasks requiring higher levels of physical effort (Bhambhani et al., 2006; Mandrick et al., 2013; Derosière et al., 2014). Mandrick and colleagues evaluated force variability and cognitive task performance in a dual-tasking paradigm (Mandrick et al., 2013). Healthy participants performed an isometric grip task at 15% and 30% of maximal effort while simultaneously performing a mental arithmetic task. Compared to the 15% condition, the 30% condition yielded greater variability of grip force, poorer performance on the mental task, and greater activity in the prefrontal cortex. The latter finding is also consistent with other recent studies which demonstrated that prefrontal activity increases in parallel with higher levels of force output (Bhambhani et al., 2006; Derosière et al., 2014). There are at least two major conditions where the detrimental effects of physical effort on automaticity may manifest as poorer mobility function: weakness and obesity. Both weakness and obesity increase the physical effort needed to perform mobility tasks (Hortobágyi et al., 2003; Bragge et al., 2014), and both are common in clinical populations. Consistent with the assertion that obesity increases the attentional demands of postural control, Mignardot and colleagues showed that both postural sway and auditory reaction time were worse during unipedal stance for obese vs. non-obese participants (Mignardot et al., 2010). A search of the literature revealed no studies to date that have examined the effects of weakness on automaticity of walking.

### Pain

Pain has been linked to mobility deficits (Karttunen et al., 2012; Demura et al., 2014), and may disrupt the automaticity of walking through a number of mechanisms. One is intentional avoidance of pain (de Gier et al., 2003) in which an individual may consciously adjust his movements in order to minimize the occurrence of pain. In the context of walking, this would imply the use of an executive locomotor control strategy. Another mechanism may be interference between the neural control pathways for pain and automaticity. Prior studies have demonstrated that pain exerts strong inhibitory influences on motor activity through spinal and cerebral mechanisms (Le Pera et al., 2001; Farina et al., 2003; Don et al., 2008). The functional implications of pain have been examined using dual-task paradigms. Most of the existing research has been conducted on patients with low back pain. A study by Sipko and colleagues examined the influence of a hard vs. soft surface on postural control in patients with low back pain. They report that patients with higher pain levels exhibit deficits in the postural adaptability to surface compliance and greater use of executive control for balance (Sipko and Kuczyński, 2012). A number of studies have found that abnormal trunk and postural control during walking or standing balance tasks in patients with low back pain is further compromised by the addition of a cognitive task (Lamoth et al., 2008; Sherafat et al., 2014). In contrast, others have reported that postural sway and trunk stiffness for a seated postural control task in patients with low back pain were improved (Van Daele et al., 2010) by the addition of a cognitive task. A possible reason for these apparently discrepant findings may be the difficulty level of the coordination task (Van Daele et al., 2010; Sherafat et al., 2014). Additional research is needed to better understand the link between pain and automaticity during walking, including in conditions other than low back pain.

### State Anxiety

Anxiety can increase the attention that is dedicated to locomotor control, which implies a shift from automaticity to an executive control strategy (Gage et al., 2003). One form of anxiety that is applicable to walking is the fear of falling, which is common in neurologically compromised and elderly individuals. Heightened anxiety due to the fear of falling has been linked to abnormal performance on tasks of balance and gait (Adkin et al., 2002; Brown et al., 2002; Carpenter et al., 2004; Hadjistavropoulos et al., 2012). For instance, Brown and colleagues compared walking performance on the ground vs. on an elevated walkway (Brown et al., 2002). The elevated walking condition had a variety of effects on the walking pattern including altered spatiotemporal variability, joint kinematics and neuromuscular activation. Similarly, concerns about self-presentation may pose another form of anxiety for individuals with movement disorders. Self-presentation refers to a person’s attempt to monitor and control how he is perceived by others (Leary, 1995). A person with an impaired gait pattern who feels judged by others may devote heightened attention to the control of walking in a conscious attempt to move more normally. Preliminary evidence from Lamarche and colleagues suggests that self-presentational concerns may be detrimental to balance performance and fall risk (Lamarche et al., 2014).

### Use of Assistive Devices

Assistive devices such as canes and walkers are vital for facilitating independent functioning in individuals with a variety of walking-related impairments. Improvements in walking ability with the use of assistive devices can be due to reduced demand for limb loading and improved balance/orientation due to somatosensory feedback from the hands (Ely and Smidt, 1977; Bateni and Maki, 2005). These benefits would be expected to improve the automaticity of walking, based on the evidence reviewed earlier in this section. Yet accumulating evidence suggests that the use of an assistive device can actually increase the executive demands of walking due to the need to control movement of the device in addition to movement of the limbs (Bateni and Maki, 2005). Multiple studies have used dual-tasking paradigms and found that walking with a cane or rolling walker slowed the performance of a reaction time task (Wright and Kemp, 1992; Wellmon et al., 2006). In some cases, assistive devices may even contribute to the occurrence of injurious falls (Stevens et al., 2009). These findings highlight the importance of appropriate selection and customization of assistive devices for each patient, in order to optimize physical assistance as well as automaticity of walking.

### Biomechanical Structure

The biomechanical structure of the lower extremity, including passive elastic properties of muscle and connective tissue, are important to the coordination and efficiency of walking (Whittington et al., 2008; Zelik et al., 2014). Research in the field of engineering has demonstrated that two legged multi-jointed machines are capable of coordinated “walking” with little to no source of external power (McGeer, 1993; Collins et al., 2005). Although less complex than true human locomotion, these machines demonstrate the impressive role of biomechanical features for producing a well-organized pattern of walking. Therefore, factors that interfere with these biomechanical features may be detrimental to locomotor control. This could be particularly important in the context of clinical populations who wear rigid braces or orthoses. A search of the literature revealed a number of studies that have examined the link between orthosis stiffness and aspects of gait performance (Bregman et al., 2011; Kobayashi et al., 2013; Harper et al., 2014), but none that have directly tested the effect of biomechanical constraints on the automaticity of walking. Although these supportive devices serve an important role for the patient, advances in design and materials may be advantageous if they restore or augment the natural biomechanical features that contribute to control of walking (Takahashi and Stanhope, 2013).

### Hearing Impairment

Hearing impairment has been shown to independently influence mobility function (Chen et al., 2014). A direct link to automaticity has not yet been investigated but it is reasonable to expect that such a link could exist. Auditory information has been shown to be an important influence for modulating the steady state walking pattern. For instance, a number of studies have used rhythmic auditory stimulation as a means to alter the spatiotemporal parameters of gait in patients with Parkinson’s disease, stroke, and other neurological disorders (del Olmo and Cudeiro, 2005; Hausdorff et al., 2007; Kadivar et al., 2011; Wittwer et al., 2013; Rodger et al., 2014). These studies generally report a positive influence, such as one by Hausdorff and colleagues who reported a more automatic movement pattern with less stride-to-stride variability when gait was timed to a metronome (Hausdorff et al., 2007). Furthermore, evidence suggests that auditory information is an important factor causing unintentional synchronization of stepping when humans walk side-by-side (Zivotofsky et al., 2012). Based on these findings, hearing impairment has the potential to compromise automaticity of walking.

## Strategies for Rehabilitation

The findings discussed in this article lead to two primary recommendations for research that seeks to enhance automaticity of walking in people with compromised mobility function. The first is to assess automaticity as an independent outcome in rehabilitation trials, in order to evaluate treatment effects on this aspect of locomotor control. The second recommendation is to develop novel therapeutic interventions that are designed to promote recovery of automaticity, and to assess these novel interventions against current best practice. Each of these recommendations will be addressed in more detail below.

This article has previously noted that the balance between automaticity and executive control can be assessed using behavioral approaches (e.g., dual-task) and/or neurophysiological approaches (e.g., brain imaging). The choice of which assessment(s) to use will depend on the resources available in the research/clinical environment and the preferences of the evaluator. Presently only a small proportion of studies include measures of automaticity, instead relying on traditional physical performance outcomes such as walking speed and gait characteristics. Physical performance outcomes are certainly valuable to assess, but may not adequately indicate the extent to which a healthier locomotor control strategy is being developed. For example, a rehabilitation intervention that yields no apparent benefit to walking speed should not be assumed to be ineffective. It may in fact be quite effective at improving the automaticity of walking, and may yield a resultant improvement in mobility safety and independence even without substantial benefit to speed. Likewise, an intervention that is found to increase walking speed may not necessarily improve the automaticity of walking. A search of the literature did not reveal any studies that were designed to directly address this issue. While there are a number of studies that have used dual-task interventions and dual-task assessments, this study design is problematic for gauging changes in automaticity. This is because dual-task interventions, although potentially beneficial to automaticity of walking, also train the ability to concurrently perform executive control tasks. Therefore, there is a confounding influence that make it challenging to interpret whether performance gains on dual-task assessments after a dual-task intervention are caused by improvements in automaticity (e.g., spinal/brainstem locomotor control) or to a better ability to manage concurrent executive control of multiple tasks. In future studies, neurophysiological assessments such as fNIRS may be valuable in distinguishing between these distinct aspects of locomotor recovery.

There is a surprising lack of research on the topic of enhancing automaticity of walking. Like other properties of motor control, automaticity can improve through motor learning. All healthy individuals have previously learned automaticity of walking during childhood, and most have also achieved automaticity of other common motor tasks such as speaking, driving or typing. Two key ingredients of motor learning are repetition and task specificity. By repeatedly activating particular neurons in a task-specific manner, the synaptic connections between those neurons become stronger. This is one form of “activity-dependent plasticity”, and is considered a major factor in motor learning of novel tasks. Initially, executive control processes may be needed to drive appropriate task-specific patterns of neuronal firing in lower (e.g., brainstem and spinal) centers of motor control. However, with sufficient practice and motor learning, the patterns gradually become more automatic and are gradually released from executive control. Although motor learning may be more challenging for the older and/or neurologically compromised populations that are generally targeted for therapy, there is no doubt that substantial potential for motor learning still exists. For instance, even adults with significant neurological injury are able to achieve substantial gains in walking function from therapy (Dobkin et al., 2006; Nieuwboer et al., 2009; Duncan et al., 2011; Marsh et al., 2011). Whether such gains are from enhanced automaticity or some other mode of neurological recovery is still unclear. Regardless, novel rehabilitation approaches that specifically target circuits of automaticity have the potential to yield additional meaningful gains in walking recovery. What might such a rehabilitation approach look like? There are many possibilities, but the focus should be on activities and/or adjuvants that engage and upregulate the activity of the CNS circuits of automaticity. The intent is to increase the patterned activity of these circuits at all levels of the neuraxis, in order to prime them for activity dependent neuroplasticity. A number of recent studies in the literature offer examples of such an approach. Rochester and colleagues have shown that the use of auditory, visual and somatosensory cues during gait rehabilitation in patients with Parkinson’s disease increased the acquisition and automaticity (dual-tasking ability) of walking (Rochester et al., 2010). A comparable study by Yen and colleagues (Yen et al., 2014) showed similar benefit to spinal cord injured patients. That study used two types of augmented feedback, including visual feedback of actual and desired stride length and/or proprioceptive feedback using swing resistance applied to the leg. The results showed that subjects’ stride length increased in all conditions, but the increase was greater and retained longer when both the visual and proprioceptive feedback were combined (Yen et al., 2014). In addition to augmenting CNS input to drive neuroplasticity during walking, another potentially valuable approach is to use multi-modal interventions that address the variety of impairments that force an individual into using a compensatory executive locomotor control strategy. Based on the information presented in the section entitled Mechanistic Factors that Influence Automaticity, this could include patient-specific treatment of weakness, pain, state anxiety, visual impairment, hearing impairment, etc. Rather than viewing these seemingly disparate deficits as isolated problems, researchers and clinicians should consider the cumulative implications on automaticity of walking.

## Conclusion

An important conclusion that can be drawn from this article is that automaticity of walking is not simply a theoretical construct of locomotor control. Automaticity of walking has a neurophysiological basis, can be assessed objectively and there are distinct strategies that can be used for targeted rehabilitation. Optimal rehabilitation of automaticity will require us to view mobility function from an interdisciplinary perspective of motor control. This contrasts with impairment-specific approaches that are commonly used in research today to study and combat mobility deficits. Adopting an “automaticity perspective” of walking rehabilitation has significant potential for improving mobility across a broad spectrum of clinical populations.

## Author Contributions

DC was responsible for conceiving, designing, drafting, reviewing and approving this review article. DC is accountable for all aspects of the work.

## Conflict of Interest Statement

The author declares that the research was conducted in the absence of any commercial or financial relationships that could be construed as a potential conflict of interest.
